# The Effects of Hydrogen Peroxide on the Circadian Rhythms of *Microcystis aeruginosa*

**DOI:** 10.1371/journal.pone.0033347

**Published:** 2012-03-07

**Authors:** Haifeng Qian, Baolan Hu, Shuqiong Yu, Xiangjie Pan, Tao Wu, Zhengwei Fu

**Affiliations:** 1 College of Biological and Environmental Engineering, Zhejiang University of Technology, Hangzhou, People's Republic of China; 2 College of Environmental and Resource Sciences, Zhejiang University, Hangzhou, People's Republic of China; University of New South Wales, Australia

## Abstract

**Background:**

The cyanobacterium *Microcystis aeruginosa* is one of the principal bloom-forming cyanobacteria present in a wide range of freshwater ecosystems. *M. aeruginosa* produces cyanotoxins, which can harm human and animal health. Many metabolic pathways in *M. aeruginosa*, including photosynthesis and microcystin synthesis, are controlled by its circadian rhythms. However, whether xenobiotics affect the cyanobacterial circadian system and change its growth, physiology and biochemistry is unknown. We used real-time PCR to study the effect of hydrogen peroxide (H_2_O_2_) on the expression of clock genes and some circadian genes in *M. aeruginosa* during the light/dark (LD) cycle.

**Results:**

The results revealed that H_2_O_2_ changes the expression patterns of clock genes (*kaiA*, *kaiB*, *kaiC* and *sasA*) and significantly decreases the transcript levels of *kaiB*, *kaiC* and *sasA*. H_2_O_2_ treatment also decreased the transcription of circadian genes, such as photosynthesis-related genes (*psaB*, *psbD1* and *rbcL*) and microcystin-related genes (*mcyA*, *mcyD* and *mcyH*), and changed their circadian expression patterns. Moreover, the physiological functions of *M. aeruginosa*, including its growth and microcystin synthesis, were greatly influenced by H_2_O_2_ treatment during LD. These results indicate that changes in the cyanobacterial circadian system can affect its physiological and metabolic pathways.

**Conclusion:**

Our findings show that a xenobiotic can change the circadian expression patterns of its clock genes to influence clock-controlled gene regulation, and these influences are evident at the level of cellular physiology.

## Introduction

With the exacerbation of eutrophication and chemical pollution, water blooms are increasingly frequent in lakes around the world, which has resulted in severe environmental problems. These include the release of repugnant odors and bottom-layer anoxia, which have significant adverse impacts on aquatic environments. Cyanobacterial blooms also pose a serious health hazard to humans (via water consumption) because they release cyanotoxins, which are toxic or carcinogenic [1]. Cyanotoxins have appeared in many freshwater lakes, such as Lake Erie (North America), Lake Winnipeg (Canada), Lake Victoria (the largest of the African rift lakes), Lakes Biwa and Kasimagaura (one of Japan's largest lakes) and Lake Taihu (the third-largest freshwater lake in China) [2]. More than 60,000 incidents of intoxication per year have been reported worldwide [3], and millions of dollars have been lost [4]. Furthermore, the development of urban and industrial agriculture is rapidly accelerating the input of nutrient sources into water systems. Thus, it is difficult to control the formation of water blooms only via the input of nutrients, such as nitrogen (N) and phosphorus (P) [5]. Some studies have researched chemical algaecides that inhibit the growth of cyanobacteria. Copper Sulfate (CuSO_4_) is an effective and cheap algaecide, which was used in reservoirs, lakes and ponds. However, copper compound is difficult to biodegrade, and it is easy to accumulate in organisms' bodies or sediments. The worry is that the broad application of CuSO_4_ may cause metal compound secondary pollution. Hydrogen peroxide (H_2_O_2_) is a simple oxidizing agent by quickly releasing a single oxygen atom. H_2_O_2_ exists in natural freshwater and is photochemically generated from organic constituents present in the water under the sunlight [6]. It is reported that the natural levels of H_2_O_2_ can reach up to10^−5^ M (0.34 mg L^−1^) [6], [7]. Recently, H_2_O_2_ was demonstrated as an effective chemical algaecide to limit cyanobacterial and green algal growth [8], [9]. Our previous report also demonstrated that H_2_O_2_ inhibits *Microcystis aeruginosa* growth, and changes physiological and biochemistry process [10]. Given that H_2_O_2_ is considered to be benign to the environment, only forming water upon degradation, we selected H_2_O_2_ as a potential algaecide to research its toxic mechanism.

*M. aeruginosa* is a unicellular species of cyanobacteria that is commonly involved in the formation of blue-green algae blooms and is widely distributed in eutrophic lakes, ponds and reservoirs [11]. The members of this genus are able to synthesize microcystins (MCs), a group of monocyclic heptapeptides. MCs can cause hepatotoxic diseases and liver cancer by inhibiting the activity of protein phosphatases 1 and 2A, which are key proteins in the regulation of many eukaryotic cell cycles [12], [13]. Many physiological activities of cyanobacteria are controlled by its biological clock, which allows these activities to adapt to daily fluctuations in the environment [14]. Agrawal *et al.* [15] demonstrated that photosynthetic gene transcripts are regulated by a biological clock to achieve efficiency in *M. aeruginosa*. There are three clock genes, *kaiA*, *kaiB* and *kaiC*, in cyanobacteria, and *kaiB* and *kaiC* operate as a unit with a single promoter (independent from the *kaiA* transcript) [16]. Transcription from either the *kaiA* or *kaiBC* operon is under circadian feedback regulation, and circadian rhythms of expression are necessary to maintain normal circadian sustainability [16]. In addition, SasA, a histidine kinase that interacts with the KaiC protein, was found to be required for sustaining robust circadian rhythms [17]. Cell division, amino acid uptake, nitrogen fixation, respiration, and carbohydrate synthesis are also under circadian control in cyanobacteria [18]–[20]. More recently, Straub *et al.* [21] demonstrated that many metabolites are controlled by a circadian clock in *M. aeruginosa*.

However, little is known regarding whether xenobiotics affect cell growth, photosynthesis or other metabolic pathways by changing the circadian rhythms of the clock genes or circadian genes. In this study, we comprehensively analyzed the relationship between exogenous H_2_O_2_ treatment and the circadian rhythms of the clock genes and circadian genes in *M. aeruginosa* to determine the effect of H_2_O_2_ on its growth and the transport of these stress signals to a related metabolite.

## Results

### The effect of H_2_O_2_ on the expression patterns of the clock genes in LD-grown cultures

**Figure 1:**
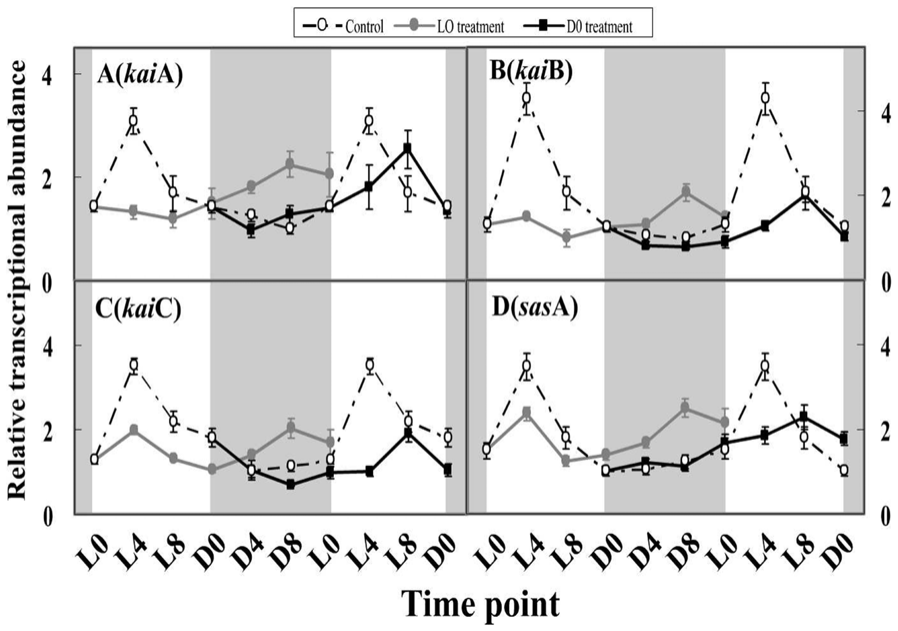
The clock gene transcripts in *M. aeruginosa* under light and dark conditions after H_2_O_2_ treatment. The shaded areas correspond to the dark period. Symbols represent mean ± SEM of triplicate cultures. The mRNA amount of all examined genes is normalized to 16S rDNA. The open circle corresponds to the control treatment; the filled circle corresponds to the L0 treatment; the filled square corresponds to the D0 treatment. A, The effect of H_2_O_2_ on the *kaiA* circadian transcript; B, the effect of H_2_O_2_ on the *kaiB* circadian transcript; C: the effect of H_2_O_2_ on the *kaiC* circadian transcript; D: the effect of H_2_O_2_ on the *sasA* circadian transcript.

The abundances of *kaiA*, *kaiB* and *kaiC* exhibit daily rhythmic patterns in the LD cycle, with peaks after 4 h of light (L4) and troughs after 4–8 h of darkness (Figure 1). A similar oscillation pattern was also observed in the daily expression of *sasA* mRNA, which reached its peak or trough at the similar time as the *kai* genes. The diurnal rhythm amplitudes (the ratios of the peak to the trough) of *kaiA*, *kaiB*, *kaiC* and *sasA* were 3.1, 4.3, 3.5 and 3.5, respectively. To determine whether H_2_O_2_ affects the circadian system, we examined the transcripts of the clock genes (*kaiA*, *kaiB*, *kaiC* and *sasA*) after H_2_O_2_ exposure at L0 and D0. In the L0 treatment, H_2_O_2_ altered the daily transcript patterns of these clock genes, and the mRNA expression of *kaiA* displayed a 16-h phase-forward and a peak time shift from L4 to D8; however, the peak mRNA transcript levels of *kaiA* did not change significantly. The daily rhythms of *kaiB*, *kaiC* and *sasA* disappeared when subjected to cosine wave analysis, as described by Wu *et al.* [22], although there was a significant daily variation in the mRNA transcript levels of *kaiC* and *sasA* (one-way ANOVA, *p*<0.05). Furthermore, the peak mRNA transcript levels of *kaiB*, *kaiC* and *sasA* decreased to 48.8%, 57.1% and 71.4% of the control (*p*<0.05), respectively. However, the amplitudes of these three genes were only 2.1, 1.5 and 1.9, respectively, which are less than those of the control.

In the D0 treatment, H_2_O_2_ altered the daily expression patterns of these clock genes by a 4-h phase delay and a peak time shift from L4 to L8. Interestingly, the abundance and amplitude of these genes also decreased, but their daily rhythms were maintained in an apparently circadian pattern in this treatment, as determined by cosine wave analysis and one-way ANOVA. The peak transcript levels of *kaiB*, *kaiC* and *sasA* decreased significantly (*p*<0.05), resulting in levels that were 46.5%, 54.3% and 65.7% of the control, respectively. The daily amplitudes of these genes were 2.5, 2.7 and 2.3, respectively.

### The effect of H_2_O_2_ on the expression of photosynthesis-related genes in LD-grown cultures

**Figure 2:**
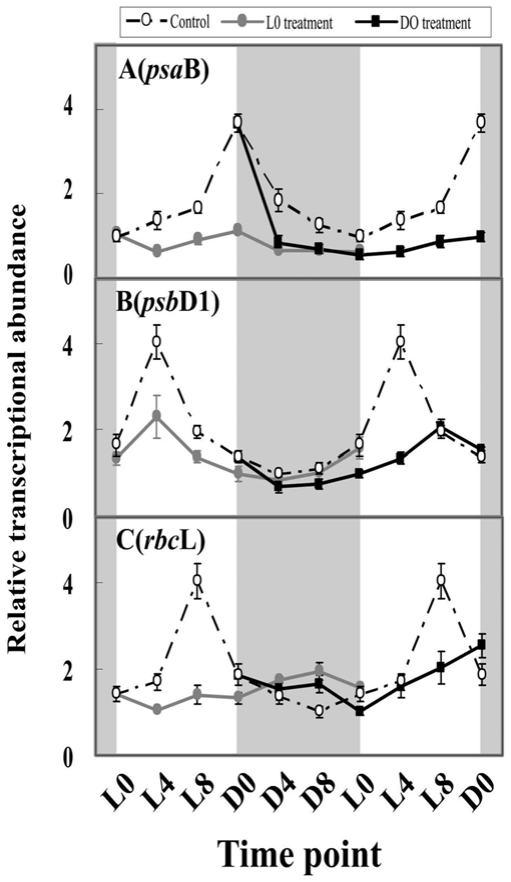
Photosynthesis-related gene transcripts in *M. aeruginosa* under light and dark conditions after H_2_O_2_ treatment. The shaded areas correspond to the dark period. Symbols represent mean ± SEM of triplicate cultures. The mRNA amount of all examined genes is normalized to 16S rDNA. The open circle corresponds to the control treatment; the filled circle corresponds to the L0 treatment; the filled square corresponds to the D0 treatment. A: The effect of H_2_O_2_ on *psaB* circadian transcript; B: the effect of H_2_O_2_ on the *psbD1* circadian transcript; C: the effect of H_2_O_2_ on the *rbcL* circadian transcript.

In cyanobacteria, oxygenic photosynthesis is a central metabolic process. To determine whether H_2_O_2_ affects the circadian system in the photosynthetic pathway, we investigated the expression of photosynthesis-related genes (*psaB*, *psbD1* and *rbcL*) after H_2_O_2_ treatment. The transcript levels of the photosynthesis-related genes were light-dependent and produced circadian expression patterns (Figure 2). The peak levels of *psaB*, *psbD1* and *rbcL* mRNA occurred at D0 (the end of light exposure), L4 and L8, respectively. The peak-to-trough ratios for *psaB*, *psbD1* and *rbcL* were 3.7, 4.0 and 4.0, respectively. H_2_O_2_ treatment at L0 significantly decreased the mRNA abundances of three photosynthesis-related genes (*p*<0.05) and changed their circadian expression patterns. H_2_O_2_ treatment reduced the peak abundances of *psaB*, *psbD1* and *rbcL* to 29.7%, 57.5 and 48.1% of the control, respectively. Their amplitudes were reduced to 33.0%, 57.5 and 47.5% of the control, respectively. The cosine wave analysis showed that H_2_O_2_ treatment caused the daily rhythms of *psaB* and *rbcL* to disappear, but H_2_O_2_ treatment did not alter *psbD1* rhythm.

H_2_O_2_ treatment at D0 also significantly decreased the transcript levels and changed the circadian patterns of the photosynthesis-related genes. The peak abundance of *psaB* decreased to 27.2% of the control treatment, and the circadian rhythm disappeared, as determined by the cosine wave analysis. The amplitude of *psbD1* mRNA expression decreased to 75% of the control, but the daily rhythm was maintained (as in the control). The amplitude of *rbcL* decreased to 62.5% of the control, and its circadian patterns exhibited a 4-h phase afterward from L8 to D0.

### The effect of H_2_O_2_ on the expression of microcystin-related genes in LD-grown cultures

**Figure 3:**
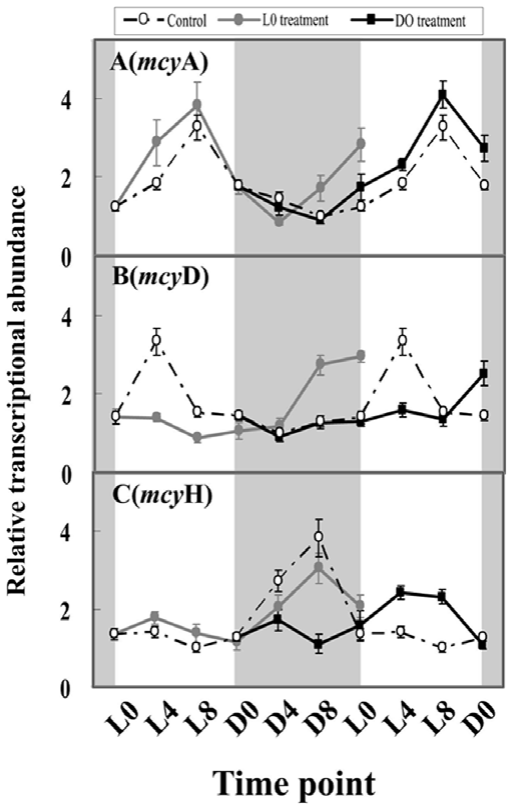
Microcystin-related gene transcripts in *M. aeruginosa* under light and dark condition after H_2_O_2_ treatment. The shaded areas correspond to the dark period. Symbols represent mean ± SEM of triplicate cultures. The mRNA amount of all examined genes is normalized to 16S rDNA. The open circle corresponds to the control treatment; the filled circle corresponds to the L0 treatment; the filled square corresponds to the D0 treatment. A: The effect of H_2_O_2_ on *mcyA* circadian transcript; B: the effect of H_2_O_2_ on the *mcyD* circadian transcript; C: the effect of H_2_O_2_ on *mcyH* circadian transcript.

The transcript levels of microcystin-related genes (*mcyA*, *mcyD* and *mcyH*) also showed circadian patterns, with peak values at L8, L4, and D8, respectively (Figure 3). H_2_O_2_ treatment at L0 did not significantly alter the peak abundances and amplitudes of these three genes with respect to the control; however, H_2_O_2_ treatment differentially affected their phases. Although H_2_O_2_ treatment at L0 did not change the daily expression patterns of *mcyA* and *mcyH*, it immediately suppressed the increase in *mcyD* mRNA levels and, thus, greatly delayed the peak phase by 20 h (from L4 to L0). H_2_O_2_ treatment at D0 also did not affect the peak abundance and phase of *mcyA*, but it greatly influenced the expression levels of *mcy*D and *mcyH*. H_2_O_2_ treatment at D0 significantly delayed the peak phases of *mcyD* and *mcyH* mRNA expression by 8 h and 12 h, respectively, with respect to the control.

### The effect of H_2_O_2_ on physiological function in LD-grown cultures

**Figure 4:**
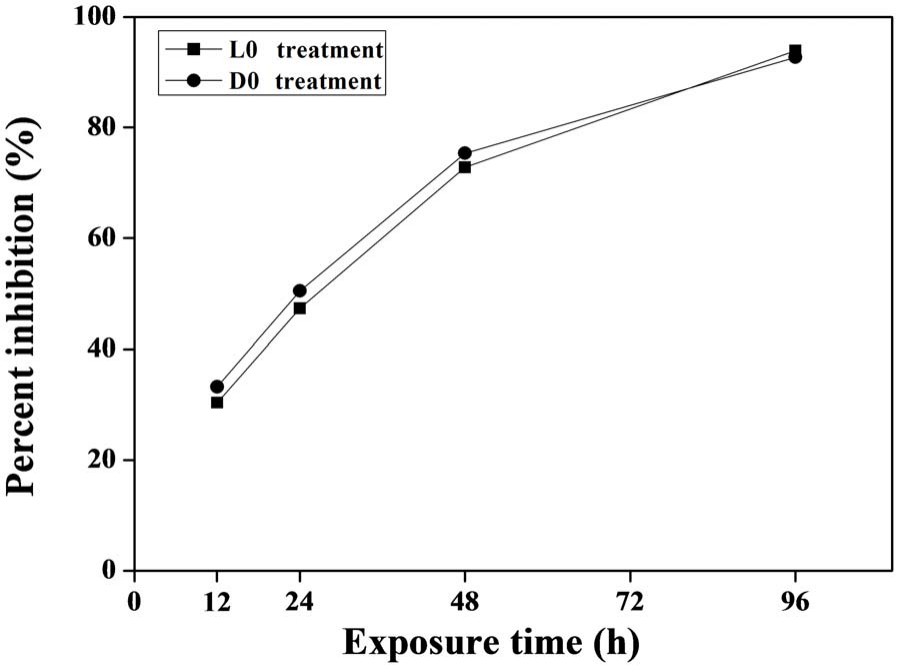
Effects of H_2_O_2_ on the inhibition of *M. aeruginosa* growth.

**Figure 5:**
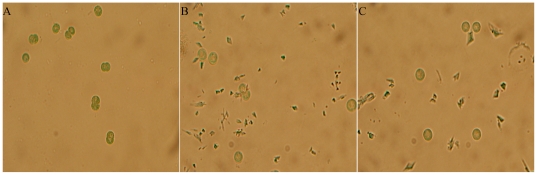
Morphology of *M. aeruginosa* cell after H_2_O_2_ exposure observed under an optical microscope (×1000). A: Control; B: L0 treatment; C: D0 treatment.

**Figure 6:**
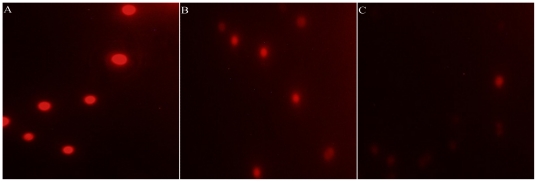
The observation of Chl a fluorescence intensity after H_2_O_2_ exposure. A: Control; B: L0 treatment; C: D0 treatment.

**Figure 7:**
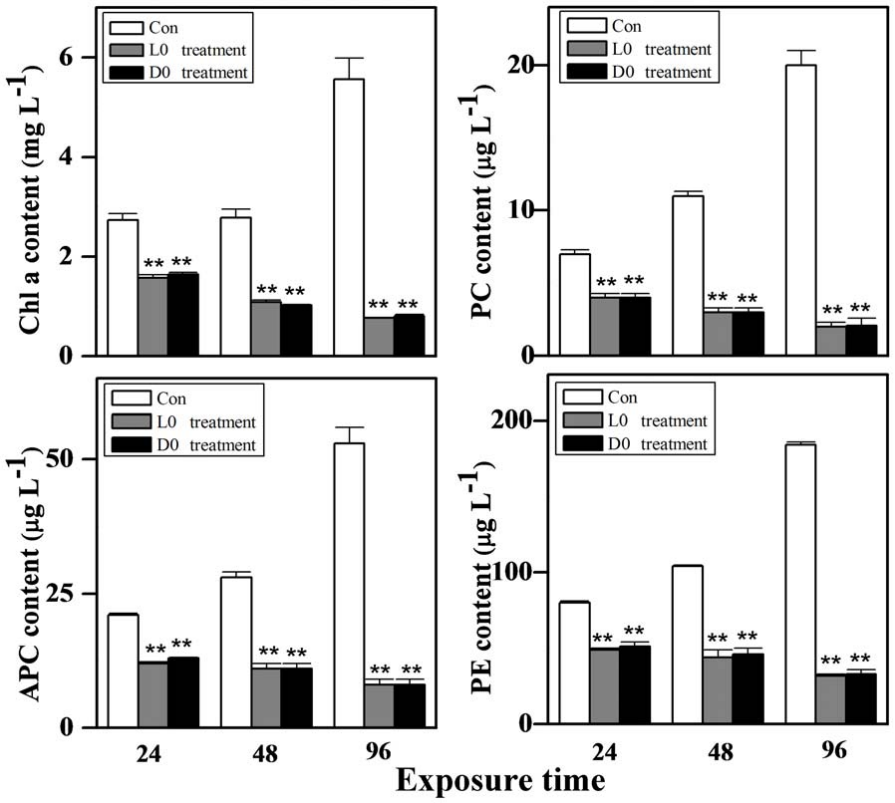
Inhibitory effects of H_2_O_2_ on Chl a, PE, PC and APC levels in *M. aeruginosa* for 24 h.

The growth of *M. aeruginosa* was significantly inhibited after H_2_O_2_ treatment at L0 or D0 point (Figure 4). The percent inhibition was exhibited in a time-dependent manner, which reached 33.2%, 50.5%, 75.3% and 92.7% after 12, 24, 48 and 96 h of H_2_O_2_ exposure at L0 point, respectively. Similar inhibitory effects of H_2_O_2_ exposure on *M. aeruginosa* growth at D0 point were also displayed. By observing cellular morphology, cyanobacterial cells were blue in normal cultural condition, but this color became weaker after H_2_O_2_ treatment, suggesting that the cells were bleached (Figure 5). Chlorophyll a (Chl a) excites optimally at 460 nm (blue light) and emits at 685 nm (red light), this fluorescence is directly proportional to the concentration of Chl a. Therefore, we analyzed the fluorescence intensity of Chl a and found that it was stronger in the control than that in the L0 or D0 treatment (Figure 6), indicating that the content of Chl a decreased after H_2_O_2_ exposure. We also quantified several pigments content, and found that the content of Chl a, phycocyanobilin (PC), allophycocyanin (APC) and phycoerythrin (PE) decreased after H_2_O_2_ exposure at L0 point. The lowest levels of them were 13.9%, 10%, 15.1% and 17.4% of the control, respectively. Similar phenomenon was also displayed after H_2_O_2_ exposure at D0 point (Figure 7).

**Figure 8:**
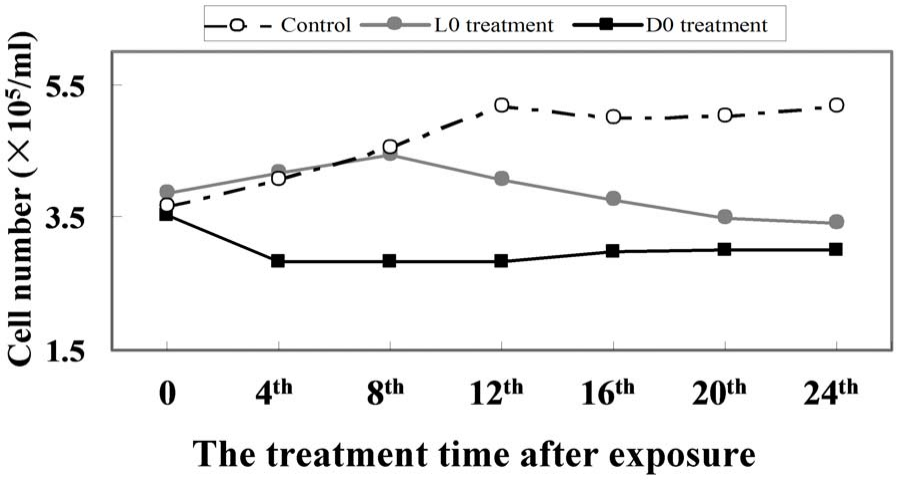
Growth of *M. aeruginosa* that was cultured with H_2_O_2_ in the first 24 h. The shaded areas correspond to the dark period. Symbols represent mean ± SEM of triplicate cultures. The open circle corresponds to the control treatment; the filled circle corresponds to the L0 treatment; the filled square corresponds to the D0 treatment.

**Figure 9:**
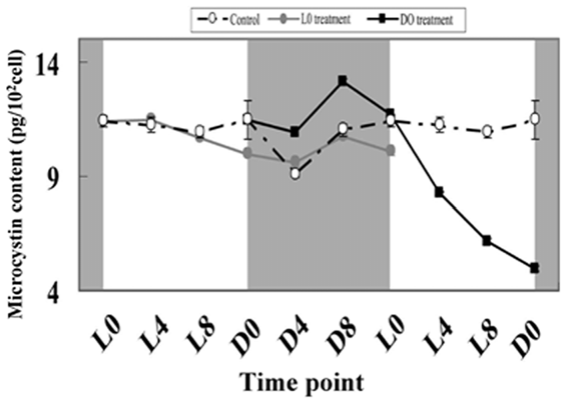
The intracellular microcystin content of *M. aeruginosa* under light and dark conditions after H_2_O_2_ treatment. The shaded areas correspond to the dark period. The symbols represent the mean ± SEM of triplicate cultures. The filled circle corresponds to the L0 treatment; the open circle corresponds to the control treatment; the filled square corresponds to the D0 treatment.

**Figure 10:**
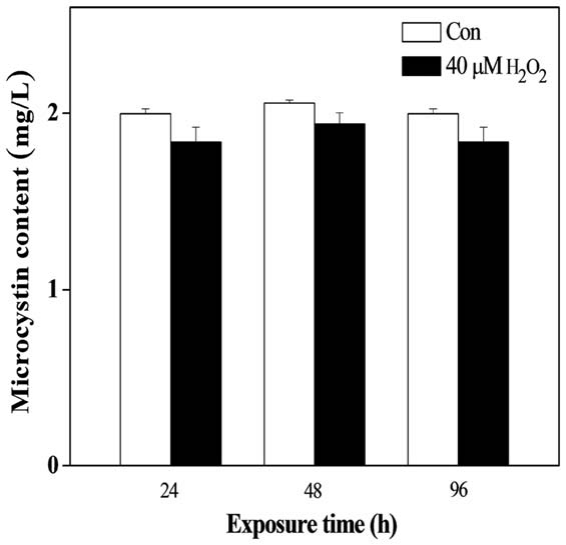
The change of microcystin content in water after oxidation by H_2_O_2_ for various time periods.

We recorded the change of cell numbers in the first 24 h exposure time, and found that *M. aeruginosa* growth was very rapid under normal conditions, cell numbers increased from 3.7×10^5^/mL to 5.2×10^5^/mL; but it slowed or decreased after H_2_O_2_ treatment at L0 and D0, cell number was from 3.8×10^5^/mL to 3.4×10^5^/mL at L0 point, and 3.6×10^5^/mL to 3.0×10^5^/mL at D0 point (Figure 8). The intracellular microcystin (MC) content fluctuated in the control group, remaining stable (approximately 11.4 pg/10^2^ cells) during the light period, declining shortly after the lights were turned off. The lowest MC content appeared at D4 point, and then returned afterwards (Figure 9). H_2_O_2_ treatment at L0 significantly reduced the MC content at D0 and caused the daily rhythm of the MC content to disappear, as determined by the cosine wave analysis. In the D0 treatment, the MC content showed a little increase during the dark period, followed by a decrease in the subsequent light period, and the lowest level was approximately 5.0 pg/10^2^ cells, which is only half of the control. The results of the cosine wave analysis showed that the daily rhythm of the MC content had also disappeared. We had suspected that H_2_O_2_ may directly oxidize MC and cause a decrease in MC content. To test this proposal, we added 50 µM H_2_O_2_ to a 2 mg/L MC solution and found that the level of MC did not significantly decrease after 24 to 96 h of exposure (Figure 10). Thus, MC is not oxidized by H_2_O_2_.

## Discussion

Water blooms are one of the top environmental issues in the world. *M. aeruginosa* is a common bloom-forming cyanobacteria in freshwater ecosystems and is of primary concern because of its wide distribution and secretion of toxic secondary metabolites, such as microcystins. Cyanobacteria are the only known bacterial species that display circadian rhythms [14], exhibiting self-sustaining oscillations with an approximately 24-h day-night cycle. These expressions are thought to be indicators of a pacemaker that controls various aspects of physiology and metabolism to adapt to daily environmental fluctuations. In cyanobacteria, approximately 30% to 80% genes exhibit robust oscillating expression profiles, and most of these genes are involved in physiological and metabolic processes [23], [24]. Using DNA microarrays, Straub *et al.* [21] demonstrated that more than 25% of the genes involved in many metabolic pathways in *M. aeruginosa* exhibit significant rhythmic changes in their transcript abundances. Their results revealed that many of the physiological and metabolic activities that occur in cyanobacteria, such as cell division, nitrogen fixation, photosynthesis, carbon uptake and the biosynthesis of secondary metabolites, are controlled by circadian rhythms.

Hydrogen peroxide treatment is considered to be more environmentally friendly than the traditional methods for cyanobacterial removal [8], [9]. Hydrogen peroxide can inhibit the growth of *M. aeruginosa* by blocking the transcription of photosynthesis-related genes or destroying photosynthetic pigments [10]. However, a clear mechanistic link among the clock genes, cellular physiological and metabolic rhythms and H_2_O_2_ (or other xenobiotics) has not been elucidated. Understanding their circadian is essential for determining the relationship between the circadian clock and the observed physiology in *M. aeruginosa*. Here, we first used real-time PCR to analyze the effect of H_2_O_2_ treatment on the circadian expression of the clock genes, circadian genes (three photosynthesis-related genes and three microcystin-related genes) within a light-dark cycle.

Since Liu *et al.* [25] introduced bacterial luciferase structural genes (*luxAB*) as a reporter of circadian gene expression in cyanobacteria, many researchers have used this method to investigate the circadian rhythms of circadian genes. Ishiura *et al.* [16] demonstrated that the transcript levels of *kaiA* and *kaiBC* in *Synechococcus* exhibit circadian rhythms, and both bioluminescence peaks occur after 9 to 12 h under LL conditions. However, bioluminescence reporters may exhibit a time lag due to transcription, translation and post-translation processes [26]. Straub *et al.* [21] used DNA microarrays and demonstrated that the transcription patterns of the *kaiB*, *kaiC* and *sasA* genes are similar, with a peak within 3 h after light exposure. In this study, the expression peaks of these clock genes appeared after 4 h of light exposure in the LD cycle. These results are similar to those found in previous studies of circadian rhythms in cyanobacteria [27], [28]. This study also observed that H_2_O_2_ treatment affected the rhythms of the clock genes. It is known that the circadian rhythm generated by the clock genes is output to downstream target genes via an unknown molecular mechanism [14]. Therefore, we analyzed the circadian rhythms of target genes (physiological-related genes, including three photosynthesis-related genes and microcystin-related genes) after H_2_O_2_ treatment.

Photosynthesis-related genes are demonstrated to transcript mRNA rhythmically for the highest efficient photosynthesis. For example, the *psbA2* gene, which encodes the D1 protein homolog of photosystem II, reaches its maximum transcript levels after 6 h of light exposure [15]. Stöckel *et al.* [23] demonstrated that most of the genes encoding the subunits of photosystem I and II are maximally expressed during light exposure and minimally expressed in the dark. In this study, we selected three photosynthesis-related genes, *psaB*, *psbD1* and *rbcL*, and found that the transcripts of these three photosynthesis-related genes displayed maximum transcript abundances in the light cycle and minimum transcript abundances in the dark cycle under normal conditions. These results also demonstrated the opinion that the circadian clock could precisely regulate photosynthetic genes to be active during day and promote organism growth in unicellular cyanobacteria [29]. This consideration was also confirmed by the growth curve of *M. aeruginosa*, which *M. aeruginosa* grows quickly in the light time, but retards in the dark time (Figure 8). The change of the rhythms of the photosynthesis-related genes after H_2_O_2_ treatment could decline solar energy utilization, and affect the synthesis of carbohydrate and high energy molecules (ATP and NADPH), which are the necessary substances for cyanobacterial growth. Therefore, this rhythmical disturbance of the photosynthesis-related genes responded in the physiological level, such as the retardation of cyanobacterial growth (Figure 8), supporting the opinion again that circadian rhythm might be important for optimal growth [30], [31].

The study also analyzed the transcripts of the microcystin-related genes, and demonstrated their circadian patterns under LD conditions. Transcript levels of microcystin biosynthesis genes (*mcyA* and *mcyD*) reached their maxima after 4–8 hours of light exposure, while the peak levels of *mcyH*, the microcystin transport gene, appeared in the dark. These results are similar to those reported by Straub *et al.* [21], who demonstrated that the biosynthesis of the microcystin genes exhibit circadian rhythms and display their maximum transcript abundance after switching to light exposure. It displayed that microcystin synthesis could be kept in step with photosynthesis, because this process need some substrates related to photosynthesis, while microcystin transport might be taken place in the dark by utilizing ATP produced at degradation of carbohydrate. To verify the rhythm of microcystin at a physiological level, we measured the microcystin content per cell under LD conditions and found that MC content was high during the light cycle and low during the dark cycle, in agreement with the rhythms of the microcystin synthesis-related gene transcripts. These results are similar to the results found by Wiedner *et al.* [32] in *Microcystis* strain PCC 7806 and Bittencourt-Oliveira *et al.* [33] in *Microcystis* spp. After H_2_O_2_ treatment, the circadian of microcystin-related genes transcripts were affected differentially, and MC content and fluctuations were changed, which shows that some physiologies are subjected to the circadian control of circadian genes.

It is very common phenomenon that organisms from bacteria to mammals use circadian clock system to adapt to daily environmental changes. The signals from the environment affect oscillator circadian rhythm by changing clock genes transcript, which in turn regulates various cellular activities, such as transcription [34], [35]. In eukaryotic species, diurnal circadian rhythm has been studied in more detail than in prokaryotic species. Reports have demonstrated that exo-factors (such as food, light) affect circadian rhythms in animals [36], [37]. This same phenomenon has also been observed in plants. Kotchoni *et al.* [38] reported that exogenous ascorbic acid affects the flowering time of *Arabidopsis* by changing the transcript levels of the clock input genes (PHYA, PHYB, CRY1, CRY2), the oscillator gene (LHY) and the output genes (GI, CO, LFY). The prokaryotic-clock research community has still focused on the composition of the timing system and discerned how it might function. Models based on empirical observations and testable hypotheses are emerging for the mechanism that underlies cyanobacterial timekeeping [39]. The knowledge about how the circadian clock controls cellular metabolism, and how extra- and intracellular environments impact the clock, is still very limited. This study showed a kind of possibility that exogenous H_2_O_2_ inhibits *M. aeruginosa* growth by affecting the transcript levels and phases of the clock genes and circadian genes, because researchers did not find the direct relationship among the clock genes and circadian genes and the physiological response up to now. Of course, this result did not exclude the possibility that H_2_O_2_ damaged cyanobacterial cell directly and caused cell death, but it may suggest a new pathway or regulatory mechanism in cyanobacteria that mediated by the circadian clock. This study can significantly broaden our understanding of temporal regulation in a unicellular oxygenic organism.

## Materials and Methods

### *M. aeruginosa* growth conditions and harvesting protocol

*M. aeruginosa* was purchased from the Institute of Hydrobiology of the Chinese Academy of Sciences (code: 905) and cultivated in BG-11 medium. The cultures were grown at 24°C in flasks (250 mL) containing 50 mL of medium and illuminated with fluorescent lights (4000 lux) for a daily 12-h-12-h light-dark cycle (LD cycle). The cell density of culture was monitored spectrophotometrically at 685 nm (OD_685_). The regression equation between the density of cyanobacteria (Y×10^5^/mL) and OD_685_ (X) was established as Y = 34.11X+0.73 (R^2^ = 0.99). When the culture reached the middle of the exponential growth phase (approximate 3.7×10^5^/mL), H_2_O_2_ (40 µM) was added to the culture at either the 0-h light point (termed L0 treatment) or the 0-h dark point (termed D0 treatment). In the LD cycle, the concentration of H_2_O_2_ was decided based on our previous report [10]. Sample collection began after 4 h of treatment and was performed every 4 h (up to 24 h).

### RNA extraction, reverse transcription and real-time PCR analysis

Culture samples (30 mL) were collected by centrifugation for RNA isolation at each time point. The cell pellet for RNA isolation was quickly frozen in liquid nitrogen until RNA extraction. Total RNA was extracted using the RNAiso kit (TaKaRa Company, Dalian, China) following the manufacturer's instructions. 500 ng RNA was reverse transcripted into cDNA according to the reverse transcriptase kit (Toyobo, Tokyo, Japan). Real-time PCR was carried out using an Eppendorf MasterCycler® ep RealPlex^4^ (Wesseling-Berzdorf, Germany). A reaction mixture for each PCR run was prepared with the SYBR® Green real-time PCR Master Mix (Toyobo, Tokyo, Japan). Final concentrations in a total volume of 10 µL were: 5 µL of 2×Master Mix (including SYBR® Green I, dNTPs, Mg^2+^, Taq DNA Polymerase, 1×ROX), 0.4 µL of 10 µM each of specific sense and anti-sense primers, and 1 µL of cDNA, 3.2 µL of ddH_2_O. The following PCR protocol was used with two steps as our previous report [10]: one denaturation step at 95°C for 1 min and 40 cycles of 95°C for 15 s, followed by 60°C for 1 min. 16Sr DNA was used as a housekeeping gene to normalize the expression profiles. Four clock genes (*kaiA*, *kaiB*, *kaiC* and *sasA*) and six circadian genes (*psaB*, *psbD1*, *rbcL*, *mcyA*, *mcyD* and *mcyH*) were selected and the primer pairs for each are listed in Table S1. For verify the efficiency of amplification determined for each primer set, dissociation curve was run following the real-time PCR, and found no other dsDNA was amplified including primer dimers, contaminating DNA, and PCR product from misannealed primer, except the targeted fragment. Agarose gel electrophoresis of PCR products also demonstrated that only targeted fragment was amplified. The relative quantification of gene expressions among the treatment groups was analyzed by the 2^−ΔΔ*C*^ *_t_* method [40], where *C_t_* is the cycle number at which the fluorescent signal rises statistically above the background.

### Microcystin analysis

Aliquots (30 mL) were centrifuged to collect the *M. aeruginosa* bodies. The aliquots were then resuspended in 300 µL of Milli-Q water to extract the microcystins using the boiling water bath method, as described by Metcalf and Codd [41]. The MC content was calculated by a standard curve, which was drafted from standard microcystin-LR (MC-LR) that was purchased from Sigma-Aldrich (CAS number: 101043-37-2, analytical standard). The microcystins were analyzed using HPLC, as described by Feng et al [42] with some modification. HPLC was performed on a Jasco LC-2000 series HPLC system (Jasco, Tokyo, Japan) equipped with C18 column (4.6 mm I.D.×250 mm, Shimadzu, Japan). The mobile phase was composed of methanol/water (6/4 by volume) plus 0.04% trifluoroacetic acid, and a flow rate of 1 mL/min was used and 20 µL of sample were injected for analysis. For detection, the circular dichroism (CD) detector was operated at 238 nm, and the column temperature was 25°C. Chromatographic data were acquired and processed with N2000 Online ChromStation software (Zhida Information Ltd. Co, Zhejiang University, China).

### Data analysis

All data are presented as mean ± standard error of the mean (SEM) and tested for statistical significance by analysis of variance (ANOVA) followed by the Dunnett's post hoc test using StatView 5.0 program. When the probability (*p*) was less than 0.05, the values were considered significantly different.

## Supporting Information

Table S1 **The sequences of primer pairs used in real-time PCR.**
(DOC)
