# Supplementary material for: The Effects of Hydrogen Peroxide on the Circadian Rhythms of Microcystis aeruginosa
Source: PLoS One. 2012 Mar 7;7(3):e33347. doi: 10.1371/journal.pone.0033347 (PMC3296681; doi:10.1371/journal.pone.0033347)
Supplement: Table S1 — The sequences of primer pairs used in real-time PCR. (DOC) [file pone.0033347.s001.doc]

**Table S1 The sequences of primer pairs used in real-time PCR.**

| Gene name | Primer | Position | GeneID |
| --- | --- | --- | --- |
| 16S rRNA | Forward:5’- GCCGCRAGGTGAAAMCTAA-3’  Reverse:5’- AATCCAAARACCTTCCTCCC -3’ | 140-369 | U03402 |
| *psa*B | Forward:5’- CGGTGACTGGGGTGTGTATG -3’  Reverse:5’- ACTCGGTTTGGGGATGGA -3’ | 348-460 | GeneID:  5865589 |
| *psb*D1 | Forward:5’- TCTTCGGCATCGCTTTCTC -3’  Reverse:5’- CACCCACAGCACTCATCCA -3’ | 109-189 | GeneID: 5864945 |
| *rbc*L | Forward 5’- CGTTTCCCCGTCGCTTT -3’  Reverse:5’- CCGAGTTTGGGTTTGATGGT -3’ | 762-889 | GeneID: 5865621 |
| *mcy*D | Forward:5’- GGTTCGCCTGGTCAAAGTAA -3’  Reverse:5’- CCTCGCTAAAGAAGGGTTGA -3’ | 10960-11257 | GeneID:  5864684 |
| *mcy*A | Forward:5’- GCCGATGTTTGGCTGTAAAT -3’  Reverse:5’- ATCCAGCAGTTGAGCAAGC -3’ | 6149-6338 | GeneID:  5864681 |
| *mcy*H | Forward:5’- GGAATGCAGGAACTGGTTGTTT-3’  Reverse:5’- GTCATTTCACGGGTTGTTTTAGG-3’ | 1262-1391 | GeneID: 5864688 |
| *kai*A | Forward:5’- CGATTGTTTGCTGGTGGTCTT -3’  Reverse:5’- TTCGTCTTGGCAGTGATGTTCT -3’ | 709-776 | GeneID:  5863990 |
| *kai*B | Forward:5’- GTCGCTGGGAACACCCCCAA -3’  Reverse:5’- CACCGGGGGAGGGAGGACTT -3’ | 807-933 | GeneID:  5863991 |
| *kai*C | Forward:5’- TGAGCGGGCGATTTTGTT -3’  Reverse:5’ - CCCTTGCGTTCCATCTCCT -3’ | 2-88 | GeneID:  5863992 |
| *sas*A | Forward:5’- ACAGCGATGGAAGCAGCGGT -3’  Reverse:5’- GGCGAACTCATCCGCATCGC -3’ | 47-180 | GeneID:  5866927 |
